# Development of a prognostic signature for esophageal cancer based on nine immune related genes

**DOI:** 10.1186/s12885-021-07813-9

**Published:** 2021-02-04

**Authors:** Zhi Zhang, Cheng Chen, Ying Fang, Sheng Li, Xiaohua Wang, Lei Sun, Guoren Zhou, Jinjun Ye

**Affiliations:** Jiangsu Cancer Hospital & Jiangsu Institute of Cancer Research & Nanjing Medical University Affiliated Cancer Hospital, 42 Bai Zi Ting Road, Nanjing, 210000 Jiangsu China

**Keywords:** Esophageal cancer, Prognostic index, Immune-related genes, TCGA

## Abstract

**Background:**

Function of the immune system is correlated with the prognosis of the tumor. The effect of immune microenvironment on esophageal cancer (EC) development has not been fully investigated.

**Methods:**

This study aimed to explore a prognostic model based on immune-related genes (IRGs) for EC. We obtained the RNA-seq dataset and clinical information of EC from the Cancer Genome Atlas (TCGA).

**Results:**

We identified 247 upregulated IRGs and 56 downregulated IRGs. Pathway analysis revealed that the most differentially expressed IRGs were enriched in Cytokine-cytokine receptor interaction. We further screened 13 survival-related IRGs and constructed regulatory networks involving related transcription factors (TFs). Finally, a prognostic model was constructed with 9 IRGs (HSPA6, S100A12, CACYBP, NOS2, DKK1, OSM, STC2, NGPTL3 and NR2F2) by multivariate Cox regression analysis. The patients were classified into two subgroups with different outcomes. When adjusted with clinical factors, this model was verified as an independent predictor, which performed accurately in prognostic prediction. Next, M0 and M2 macrophages and activated mast cells were significantly enriched in high-risk group, while CD8 T cells and regulatory T cells (Tregs) were significantly enriched in low-risk group.

**Conclusions:**

Prognosis related IRGs were identified and a prognostic signature for esophageal cancer based on nine IRGs was developed.

**Supplementary Information:**

The online version contains supplementary material available at 10.1186/s12885-021-07813-9.

## Background

Esophageal cancer (EC) is the eighth commonest cancer worldwide. The National Cancer Institute estimated 16,910 new cases and 15,910 deaths from esophageal cancer in the United States in 2016 [1]. Its incidence has risen by more than six times (1999–2008) [2]. The overall five-year survival of EC and that after esophagectomy are still poor, although great improvements have been made in treatment [3]. Squamous cell carcinoma is the most common histological type of EC [4]. Tobacco, alcohol, and malnutrition are the most associated risk factors in the development of EC [5]. Once diagnosed, EC must be accurately staged prior to the initiation of treatment. TNM (tumor, lymph node, metastasis) is a staging system based on the status of tumor invasion, lymph node, and metastasis [6]. Early-stage EC is usually treated with endoscopic surgery, advanced EC with surgery with or without chemoradiation [7].

Certain specific genes and biomarkers are needed to predict the patient’s therapeutic response and increase their survival [3]. Immune responses is critical in the tumor microenvironment. Tumor cells with genomic alterations can produce new antigens that can be recognized by the immune cells [8]. Expression of IRGs can serve as efficient biomarkers. Previous research have explored the IRGs-based prognostic features in patients with non-squamous non-small cell lung cancer [9] and papillary thyroid carcinoma [10]. However, prognostic models based on IRGs for EC remain to be elucidated.

This study investigated the clinical significance of a prognostic model based on immunogenomics.

## Materials and methods

### Data collection

The mRNA profiles and corresponding clinical information of 11 normal tissues and 160 EC samples were downloaded from TCGA (https://www.cancer.gov/) [11], which including 81 Esophagus Squamous Cell Carcinoma (ESCC) and 79 Esophagus Adenocarcinoma (EA). A set of IRGs were obtained through the Immunology Database and Analysis Portal (ImmPort) database (https://www.immport.org) [12]. A set of tumor-related TFs were obtained from Cistrome Cancer (http://cistrome.org/CistromeCancer/) [13]. CIBERSORT (https://cibersort.stanford.edu/index.php) is based on a gene expression deconvolution algorithm [14] for obtaining immune cells with differences between cancer and normal tissues.

### Identification of differentially expressed genes (DEGs)

DEGs between EC and normal tissues were identified via R software (version: × 64 3.2.1) and package Limma. The *p* value was adjusted into the false discovery rate (FDR). A value of FDR less than 0.05 and |log2(FC)| higher than 1 were considered significant.

### Identification of immune-related genes (IRGs)

DEGs overlapped with immune-related genes were obtained as the differentially expressed IRGs. Based on these IRGs, Gene Ontology (GO) [15] and Kyoto Encyclopedia of Genes and Genomes (KEGG) [16] analyses were performed with the clusterprofiler R package to explore the underlying mechanisms of these IRGs.

### Identification of prognosis-related IRGs and construction of regulatory network

Prognosis-related IRGs were identified using univariate COX regression analysis. We analyzed these prognosis-related IRGs using the package R. Then, we investigated the interaction of these IRGs and differentially expressed TFs with a threshold of *P* < 0.05. Coefficient > 0.3 was considered as positive regulation, otherwise as negative regulation. Subsequently, we constructed a regulatory network with relevant TFs and prognosis-related IRGs by using cytoscape software 3.7.1 [17].

### Construction of a prognostic model in EC based on IRGs

We constructed a prognostic model based on the results of a multivariate Cox regression analysis. Based on the median risk score, EC patients were divided into high-risk and low-risk groups. The performance of prognostic model was validated by survival analysis between groups with thresholds of *p* < 0.05 using the survival and survminer package of R. Receiver operating characteristic (ROC) analysis was performed via the survivalROC package, and the area under curve (AUC) was calculated to evaluate the efficiency of the model in predicting disease onset [18]. At the same time, we collated the patient’s clinical information and deleted the incomplete information. Finally, a total of 115 patients’ clinical information (Supplementary Table 1) were used for univariate and multiple regression analysis to determine whether the riskscore may become an independent predictor of ESCC. Association between IRG expression and clinical parameters was tested using independent t-tests, and *p* < 0.05 were considered statistically significant. Clinical survival analysis in subgroups was also conducted, and p < 0.05 was considered statistically significant.

### Verification of the prognosis-related IRGs in this model

We used the online software Oncomine (https://www.oncomine.org) to verify the IRGs. For screening, we set the following criteria: 1 “Gene: IRGs in this model”; 2 “Analysis Type: Cancer vs. Normal Analysis”; 3 “Cancer Type: Esophageal Cancer”; 4“ Clinical Outcome: Survival Status “; 5 “Data Type: mRNA”. Based on the specific binding between antibodies and antigens, immunohistochemistry can reveal the relative distribution and abundance of proteins. Using The Human Protein Atlas (THPA) (https://www.proteinatlas.org) [19], we observed the differences in key gene expression between normal and EC tissues.

### Building a predictive nomogram

To investigate the possibility of EC 1-OS and 3-OS, we established nomograms by including all independent prognostic factors identified by multivariate Cox regression analysis. The effectiveness of the nomogram was evaluated by discrimination and calibration. Finally, we plotted the curve of the nomogram by package rms of R to observe the relationship between the predicted rate of nomogram and the observed rate.

### Functional enrichment analysis

We used Gene Set Enrichment Analysis (GSEA) [20] to identify consistent differences between high-risk and low-risk groups and the associated biological processes. In screening the gene list of KEGG, *p* < 0.05 was considered statistically significant.

### Differential expression of tumor-infiltrating immune cells between high-risk and low-risk groups

Status of immune infiltration in EC patients was achieved from the dataset of CIBERSORT. Subsequently, we tested the abundance of immune cells, and its difference between high-risk and low-risk groups by using two-sample T-test.

## Results

### DEGs between EC and normal samples

**Fig. 1:**
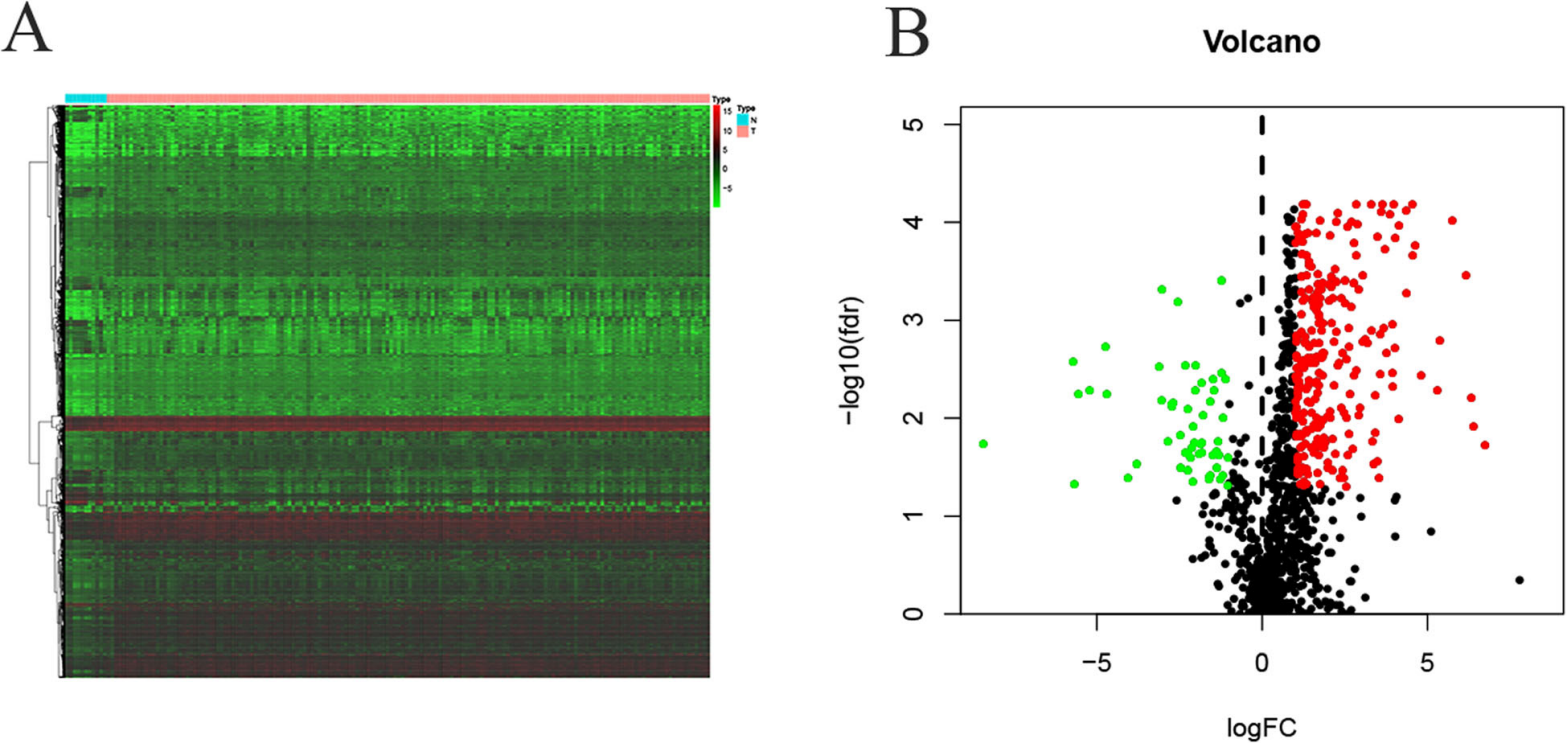
Differential expression analysis of DEGs and IRGs. **a** Heatmap of DEGs; Red plots: upregulation; Green plots: downregulation; Black plots: normally expressed mRNAs. **b** Volcano plot of IRGs; Red, green and black plots: differentially expressed mRNAs as indicated in A

The RNAseq tertiary data set of EC from TCGA included the biological information of 11 normal tissue and 160 EC samples. We identified 4094 DEGs, including 3272 upregulated DEGs and 822 downregulated DEGs. (Fig. 1a).

### Identification of IRGs

**Fig. 2:**
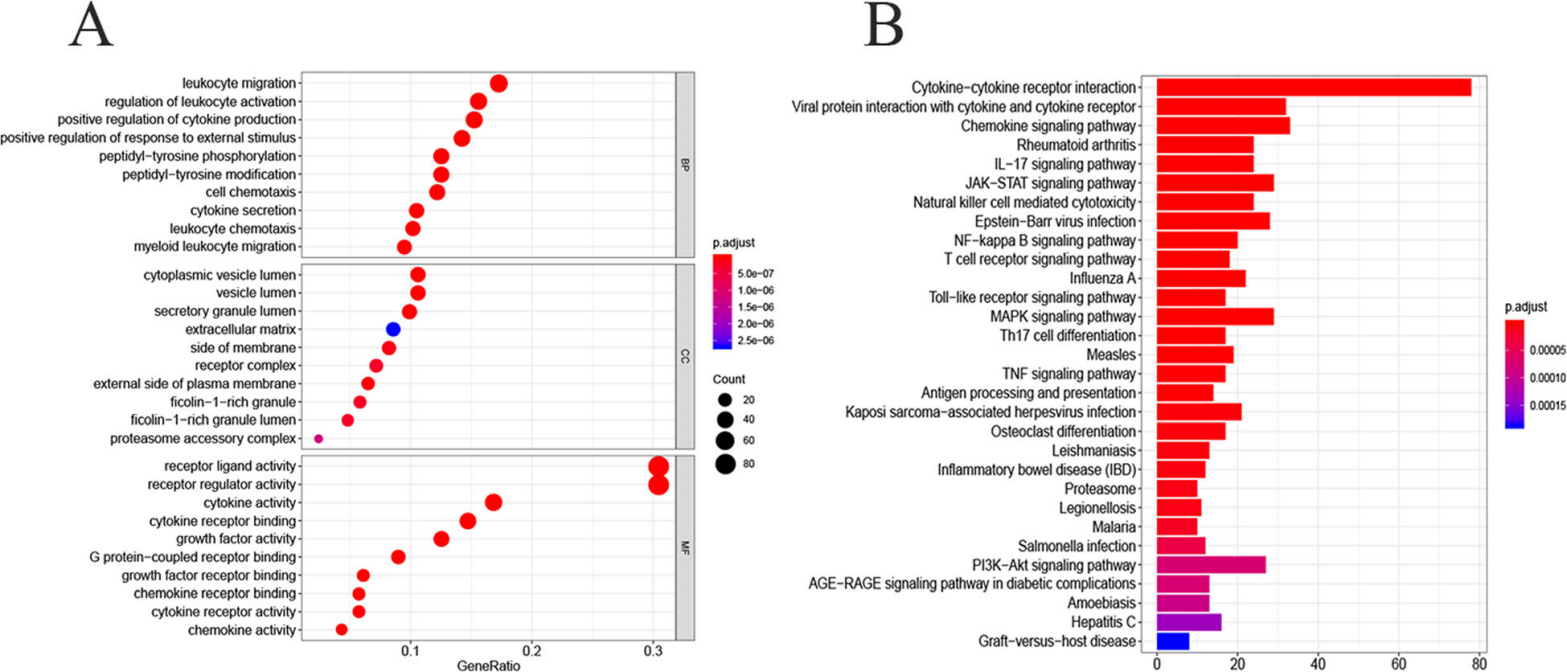
Functional enrichment analysis of differentially expressed IRGs. **a** Gene ontology analysis; the ball in the three rectangles represent biological process, cellular component and molecular function, respectively. **b** The significant KEGG pathways of IRGs

By overlapping the immune-related genes and DEGs of EC, we identified 247 upregulated and 56 downregulated IRGs, as shown in Fig. 1b. Figure 2 shows the results of functional enrichment analysis. GO analysis (Fig. 2a) demonstrated that these IRGs were most involved in leukocyte migration in Biological Process (BP), vesicle lumen in Cellular Component (CC) and receptor ligand activity in Molecular Function (MF). KEGG analysis indicated that these genes were most involved in the interaction of cytokines with cytokine receptors. (Fig. 2b).

### Survival analysis and construction of regulatory network

**Fig. 3:**
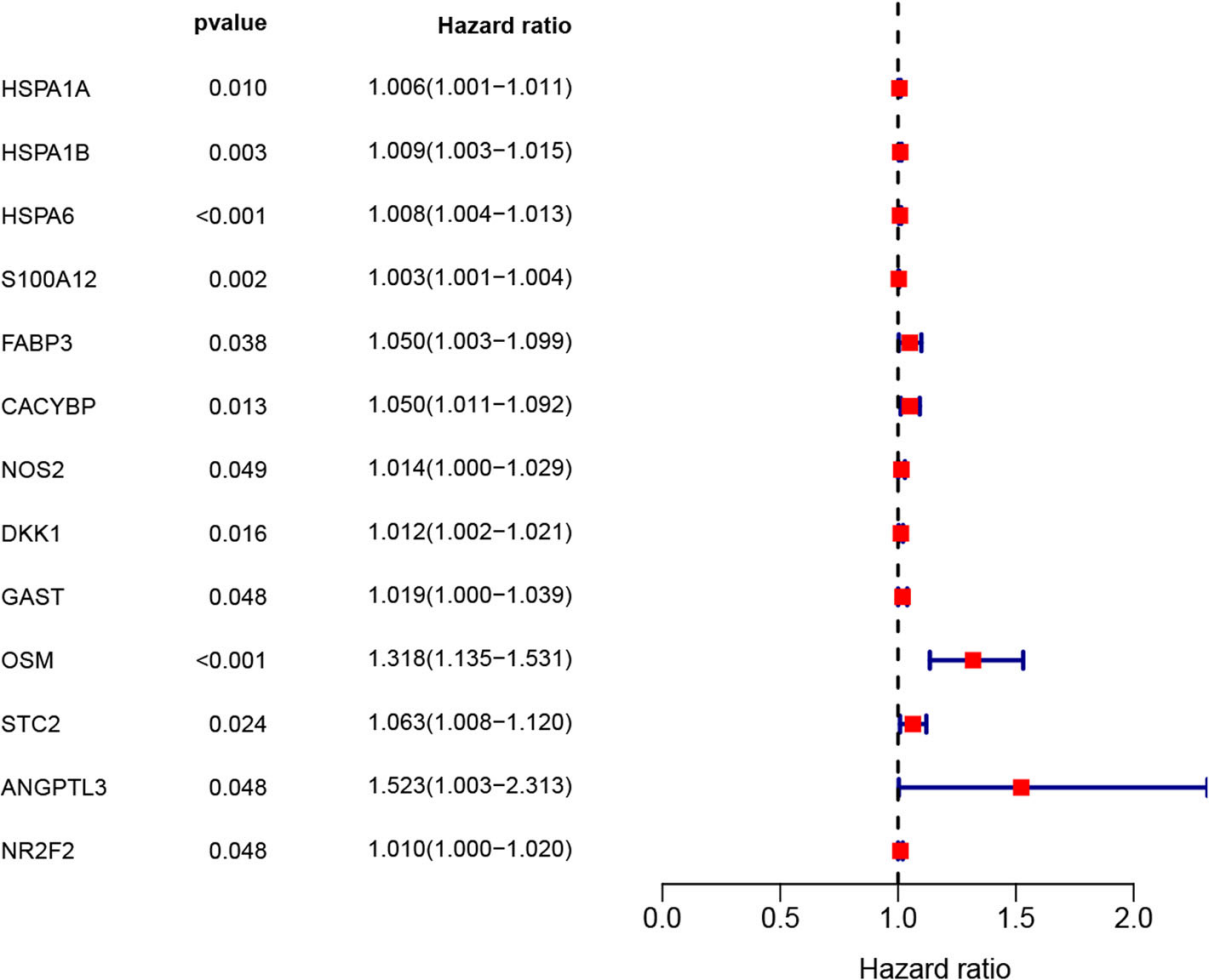
Forest plot of hazard ratios showing the prognostic values of genes, in which the unadjusted hazard ratios as well as the corresponding 95% confidence intervals are displayed

**Fig. 4:**
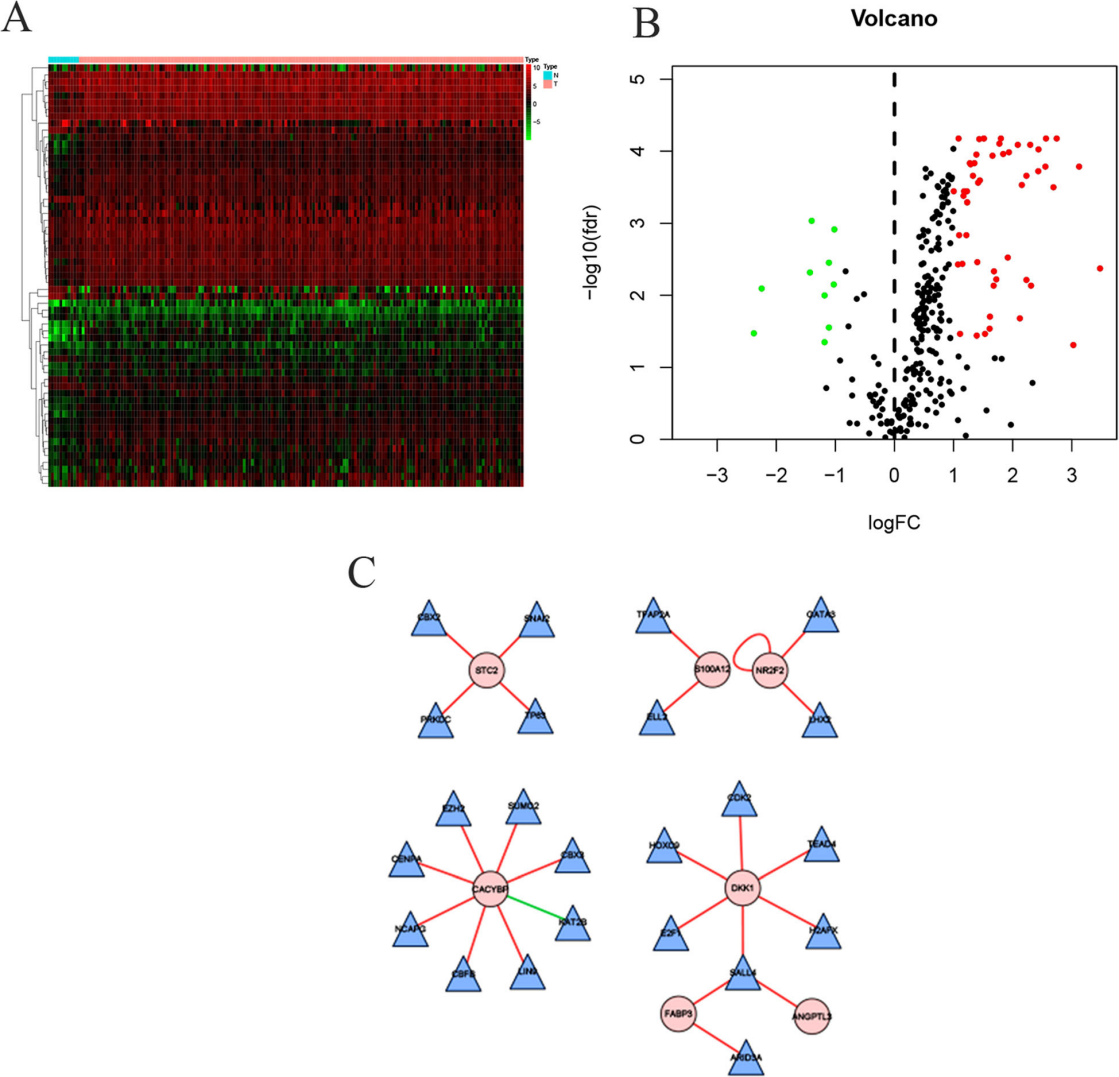
Differential expression analysis of TFs and the regulatory network. **a** Heatmap of TFs, red: upregulation; green: downregulation; black: normally expressed mRNAs. **b** Volcanic maps of TFs; red, green and black plots: differentially expressed mRNAs as indicated in A. **c** Regulatory network integrated the survival associated IRGs and differentially expressed TFs; the circles filled with pink represent the survival associated IRGs and the triangles filled with blue represent TFs

A total of 13 survival-associated IRGs were identified after integrating clinical information from TCGA via univariate COX regression, as shown in Fig. 3. After examining the expression of 318 transcription factors (TF), we found 61 with differential expressions between EC and normal samples, as shown in Fig. 4a, b. Finally a regulatory network was constructed using these survival-associated IRGs with differently expressed TFs (Fig. 4c).

### Construction of a prognostic model based on prognosis-related IRGs and external validation

**Fig. 5:**
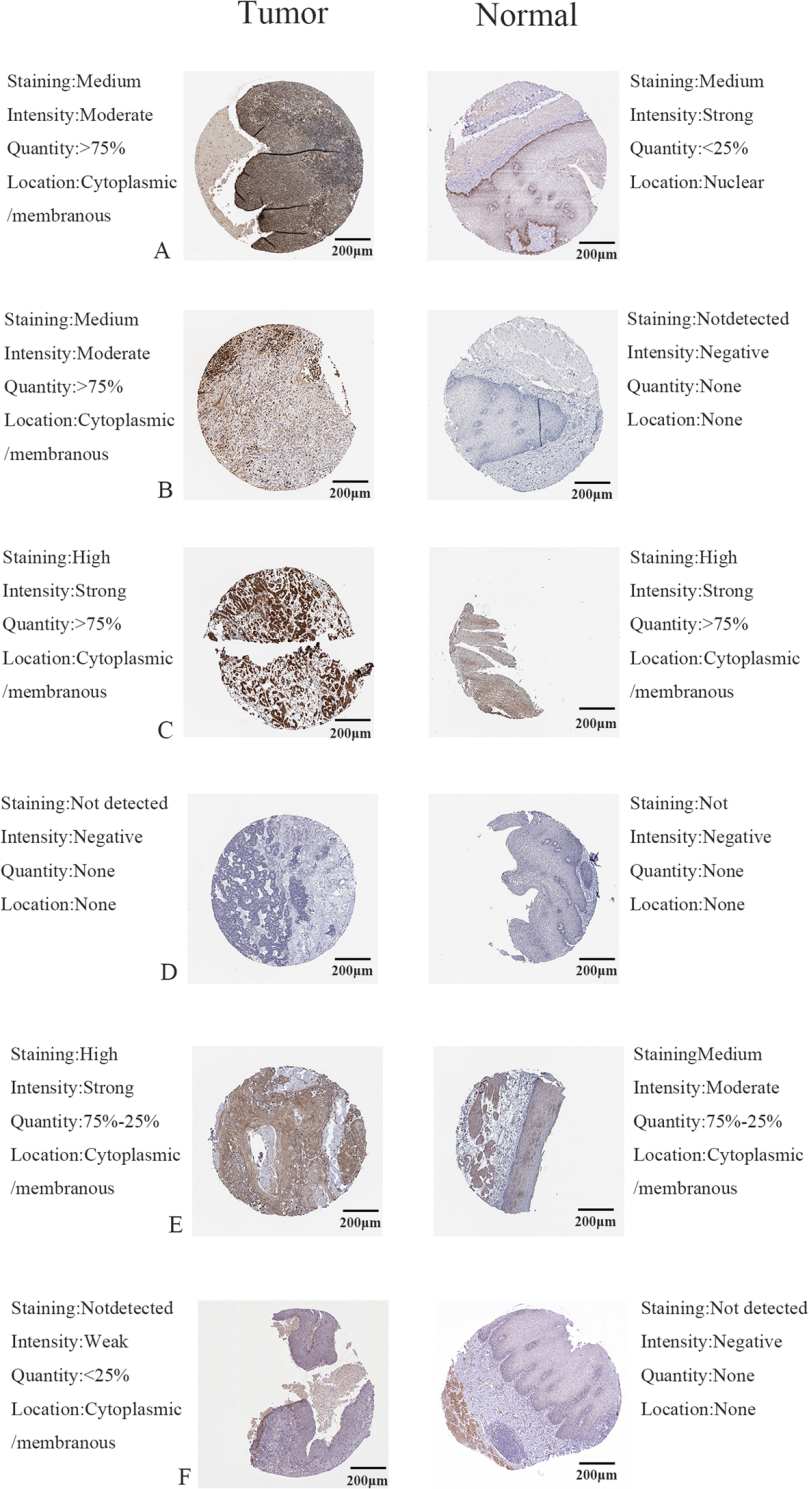
Protein expression of genes in the model. **a** protein expression of HSPA6. **b** protein expression of S100A12. **c** protein expression of CACYBP. **d** protein expression of NOS2. **e** protein expression of STC2. **f** protein expression of ANGPTL3

We constructed a prognostic model with nine prognostic IRGs based on the results of multivariate Cox regression analysis (Table 1). The formula was as follows: Risk score = expression level of HSPA6*0.006713979 + S100A12*0.003828117 + CACYBP*0.042341765 + NOS2*0.02490294 + DKK1*0.015602891 + OSM*0.207589957 + STC2*0.075574581 + ANGPTL3*0.645334283 + NR2F2*0.015710952. We further explored the protein expression of these nine prognosis-related IRGs in THPA (Fig. 5). Consistent with our results, THPA database showed that HSPA6, S100A12, CACYBP, NOS2, and STC2 in EC tissues were up-regulated, and ANGPTL3 was down-regulated compared with those in normal tissues. However, we did not find expression of DKK1, OSM and NR2F2 proteins in the database.

**Table 1:** The immune-based prognostic index model of EC

id	coef	HR	HR.95 L	HR.95H	pvalue
HSPA6	0.006713979	1.006736568	1.000468103	1.013044308	0.035133319
S100A12	0.003828117	1.003835454	1.002153991	1.005519738	7.62E-06
CACYBP	0.042341765	1.043250965	0.992392076	1.09671631	0.096819638
NOS2	0.024902941	1.02521561	1.009716012	1.040953134	0.001355421
DKK1	0.015602891	1.015725251	1.005677036	1.025873864	0.002098124
OSM	0.207589957	1.230708423	1.044607882	1.449963424	0.013076143
STC2	0.075574581	1.07850366	1.018550661	1.141985558	0.009601837
ANGPTL3	0.645334283	1.906624275	1.249904495	2.908395112	0.002741427
NR2F2	0.015710952	1.015835018	1.005558018	1.026217051	0.002459204

### Validation of the prognosis-related IRGs in the Oncomine database

We validated the reliability of the prognosis-related IRGs by using Oncomine. The databases showed that the IRGs were differentially expressed in EC and normal tissues. As shown in Supplementary Fig. 1, HSPA6, S100A12, CACYBP, NOS2, DKK1, OSM and STC2 were up-regulated, and ANGPTL3 and NR2F2 were down-regulated in EC tissues compared with those in normal tissues. We found that the results were almost consistent with our predictions.

### Validation of the prognostic capacity of the model

**Fig. 6:**
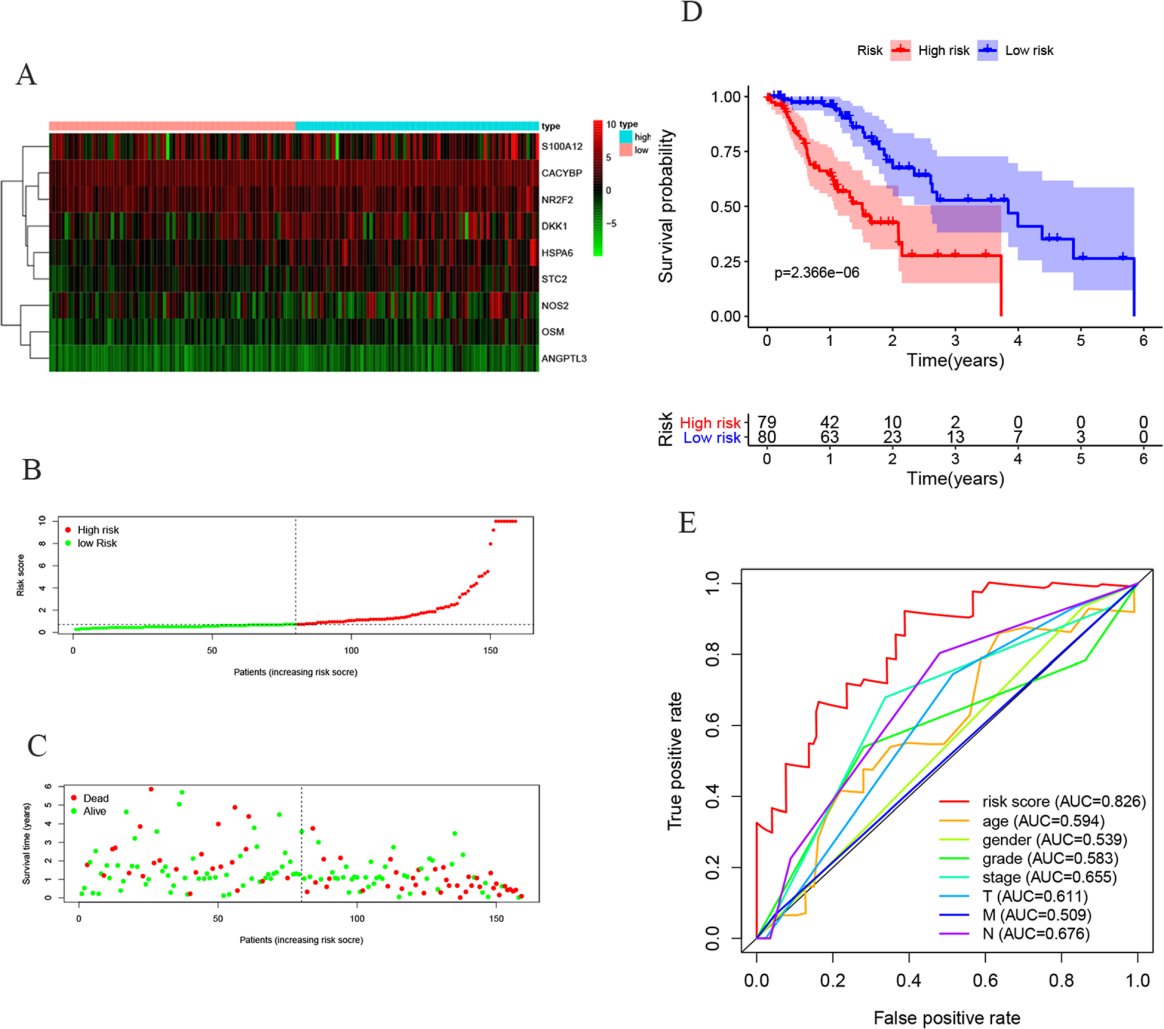
The prognostic value of prognostic index developed based on IRGs. **a** Heatmap of expression profiles of included IRGs. **b** Survival status of patients in different groups. **c** Rank of prognostic index and distribution of groups. **d** Survival analysis between the two groups.**e** ROC curve of the prognostic index model

**Fig. 7:**
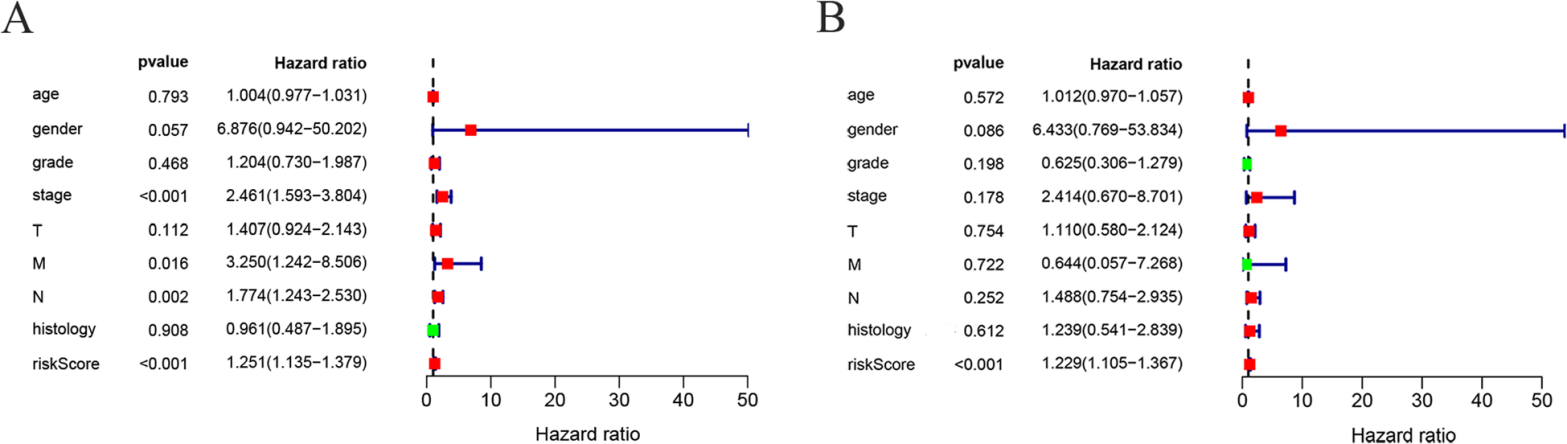
Forest plots including the risk score and other clinical parameters by univariate (**a**) and multiple regression analysis (**b**), in which the unadjusted hazard ratios as well as the corresponding 95% confidence intervals are displayed

**Fig. 8:**
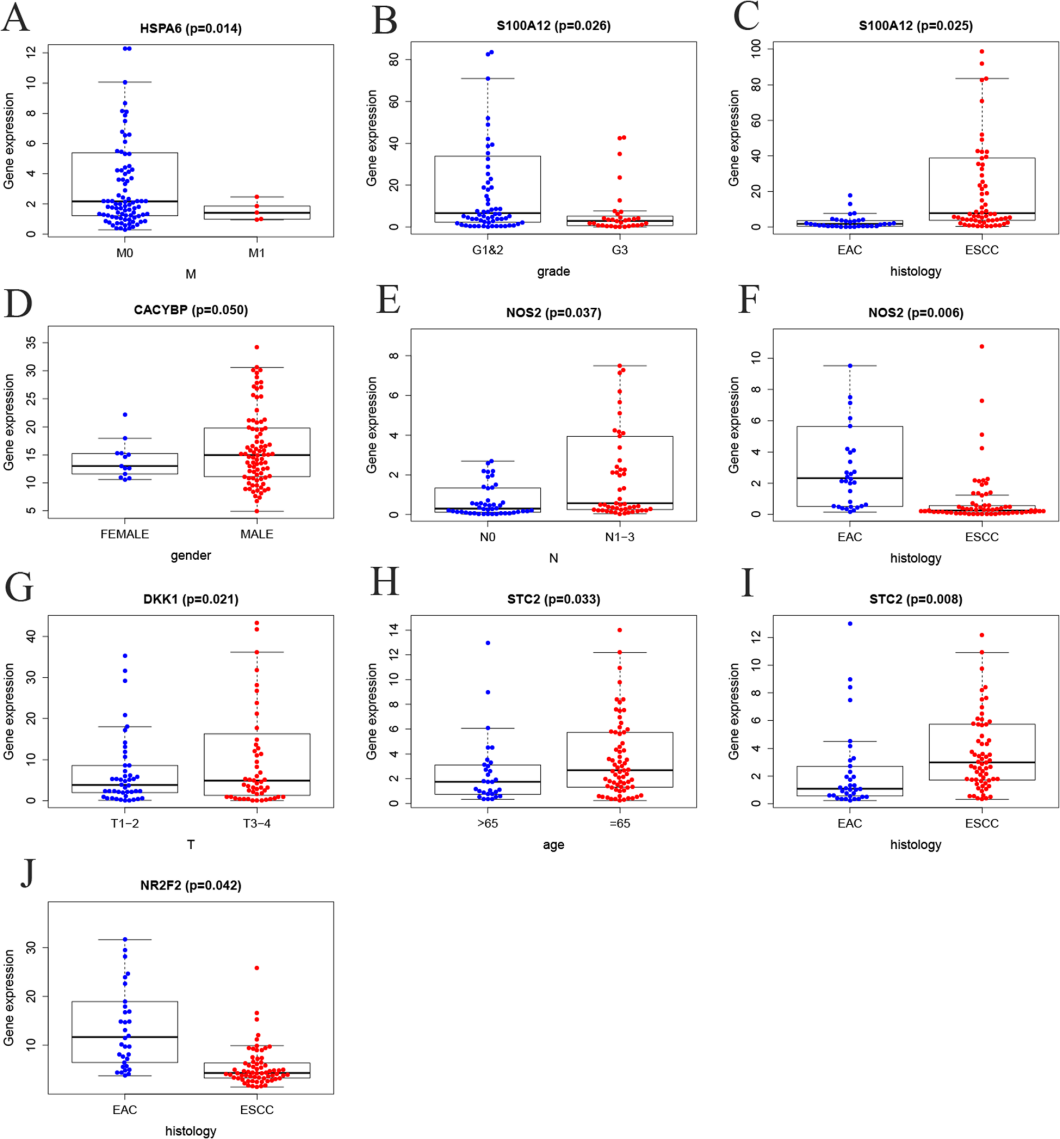
Relationships between the expressions of the immune-related genes and the clinicopathological factors.(**a**) HSPA6 and M.(**b**) S100A12 and grade.(**c**) S100A12 and histology.(**d**) CACYBP and gender.(**e**) NOS2 and N.(f) NOS2 and histology.(g) DKK1 and T.(**h**) STC2 and age.(**i**) STC2 and histology.(**j**) NR2F2 and histology

**Fig. 9:**
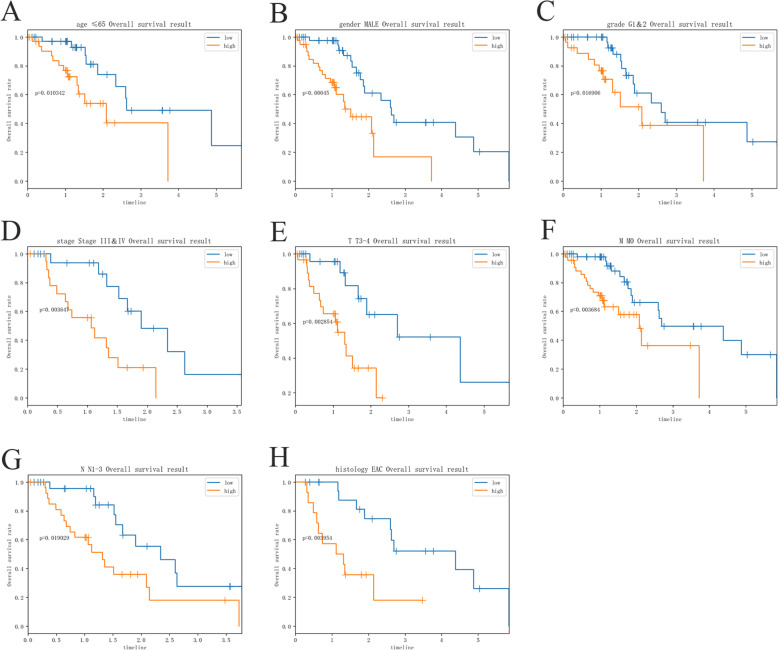
Subgroup survival analysis for patients with EC according to the prognostic index stratified by clinical factors.(**a**) age ≤ 65. (**b**) gender MALE. (**c**) grade G1&2. (**d**) stage III & IV. (**e**) T3–4. (**f**) M0. (**g**) N1–3. (**h**) histology EAC. EAC: esophageal adenocarcinoma

Patients were separated into the high-risk group and the low-risk group based on the median risk score (Fig. 6 a-c). Survival analysis showed that the survival rate in the high-risk group was remarkably lower than those in the low-risk group (*p*==2.366e− 06, Fig. 6d). The area under curve (AUC) of the receiver operating characteristic (ROC) curve was 0.826 (Fig. 6e). Compared with clinical factors (including age, gender, grade, stage and TMN), this signature showed a greater performance in predicting the prognosis of EC. At the same time, univariate and multiple regression analysis (Fig. 7a, b) showed that when other clinical parameters were adjusted, the prognostic signature may become an independent predictor. The clinical significance of included genes was also explored in this study (Fig. 8a-j). In order to assess the prognostic capacity of the model, we conducted a stratified analysis of clinical factors. Interestingly, we found that nearly the high-risk patients in subgroups of age ≤ 65(Fig. 9a), male (Fig. 9b), G1 & G2(Fig. 9c), stage III & IV (Fig. 9d), T-3-4(Fig. 9e), MO (Fig. 9f), N1–3(Fig. 9g) and EAC (Fig. 9h) were inclined to unfavorable overall survival.

### Construction and validation of predictive nomogram

**Fig. 10:**
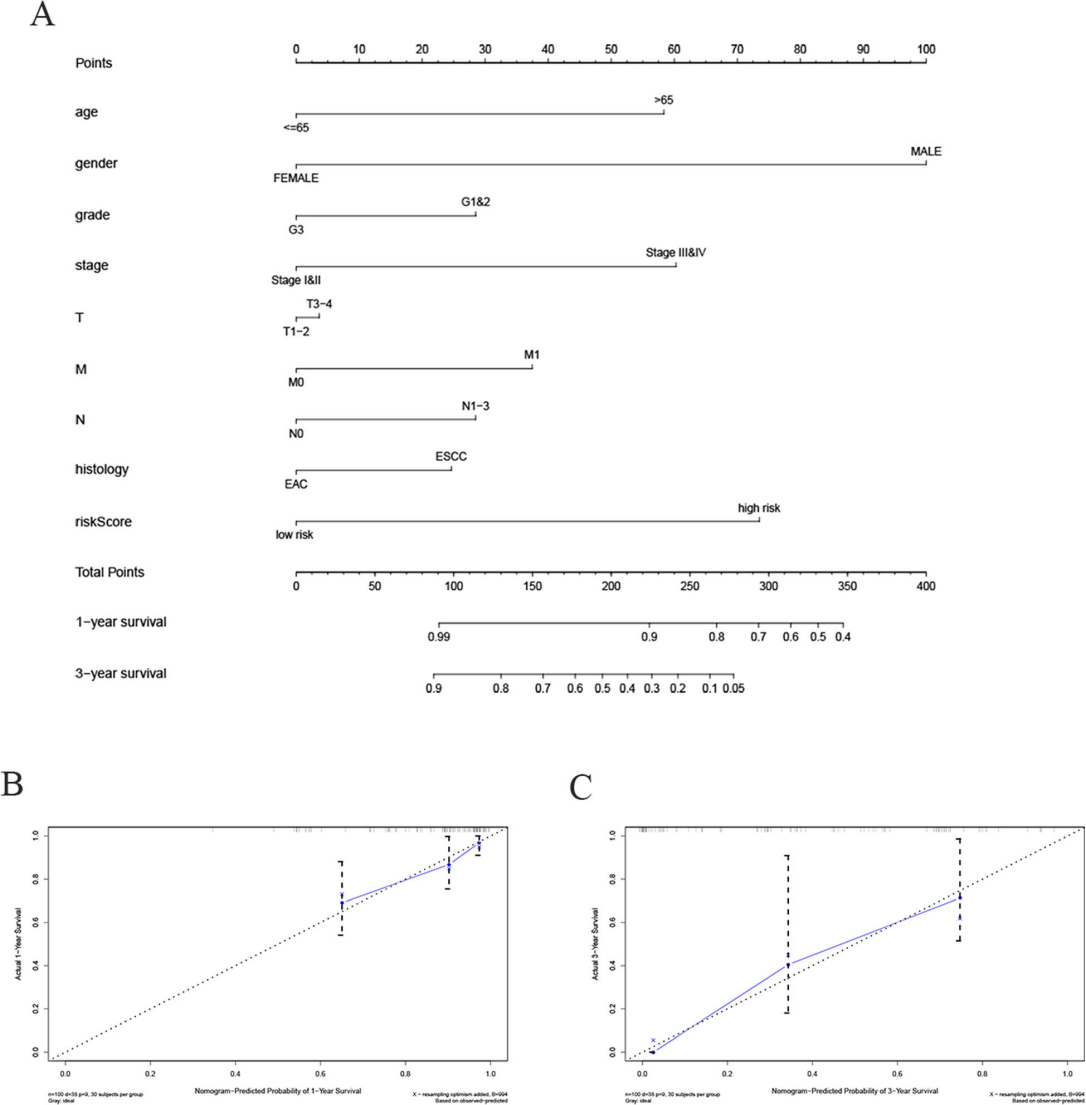
Nomogram predicting overall survival for EC patients. **a** For each patient, several lines are drawn upward to determine the points received from the predictors in the nomogram. The sum of these points is on the “total point” axis. Then a line is drawn downward to determine the possibility of 1- and 3-year overall survival of EC. **b**, **c** The calibration plot for internal validation of the nomogram. The Y-axis represents actual survival, and the X-axis represents nomogram-predicted survival

Using a number of independent prognostic factors (including age, gender, grade, stage, TMN, histology, and risk scores), we established a nomogram to predict 1-year and 3-year OS in 100 EC patients. The calibration chart showed that the nomogram might overestimate or underestimate the mortality (Fig. 10). These results suggested that the nomogram based on multiple factors can better predict short-term survival (1 year and 3 years) compared to the nomogram based on a single factor.

### Identification of related biological processes and pathways

**Fig. 11:**
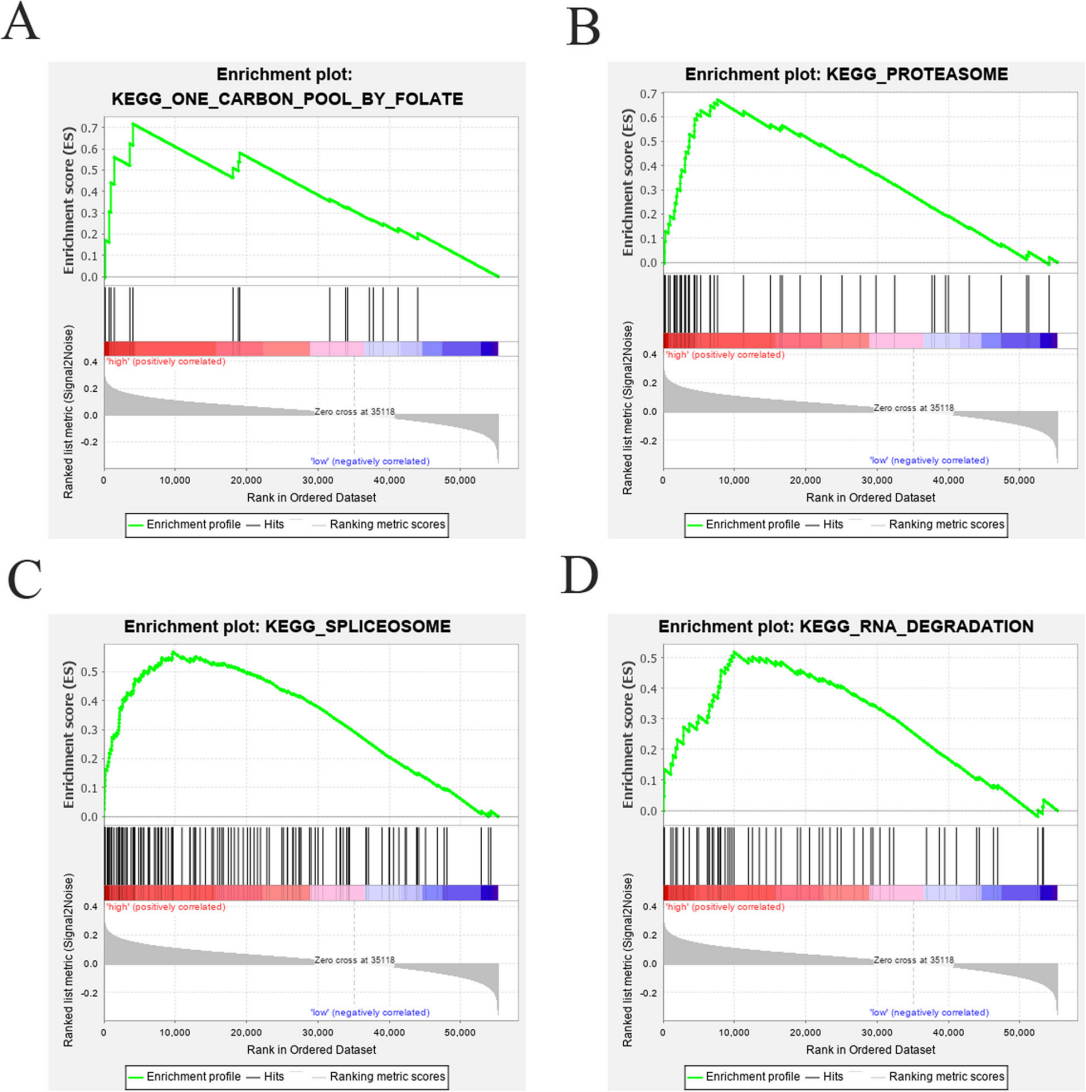
GSEA results for high and low risk differentially expressed genes in TCGA for (**a**) one carbon pool by folate, (**b**) proteasome, (**c**) spliceosome, and (**d**) RNA degradation

We employed risk score to classify the entire data set and determine the related pathways with these nine genes by using the Java software GSEA. The results showed that “one carbon pool by folate”, “proteasome”, “spliceosome” and “RNA degradation” were more abundant in the high-risk group than in the low-risk group. This suggests that in high-risk patients, the nine genes were most involved in pathways of protein degradation, RNA degradation and splicing. That is to say, patients with protein degradation, RNA degradation and splicing effects were more inclined to a poor prognosis (Fig. 11).

### Difference of tumor-infiltrating immune cells between the two risk groups

**Fig. 12:**
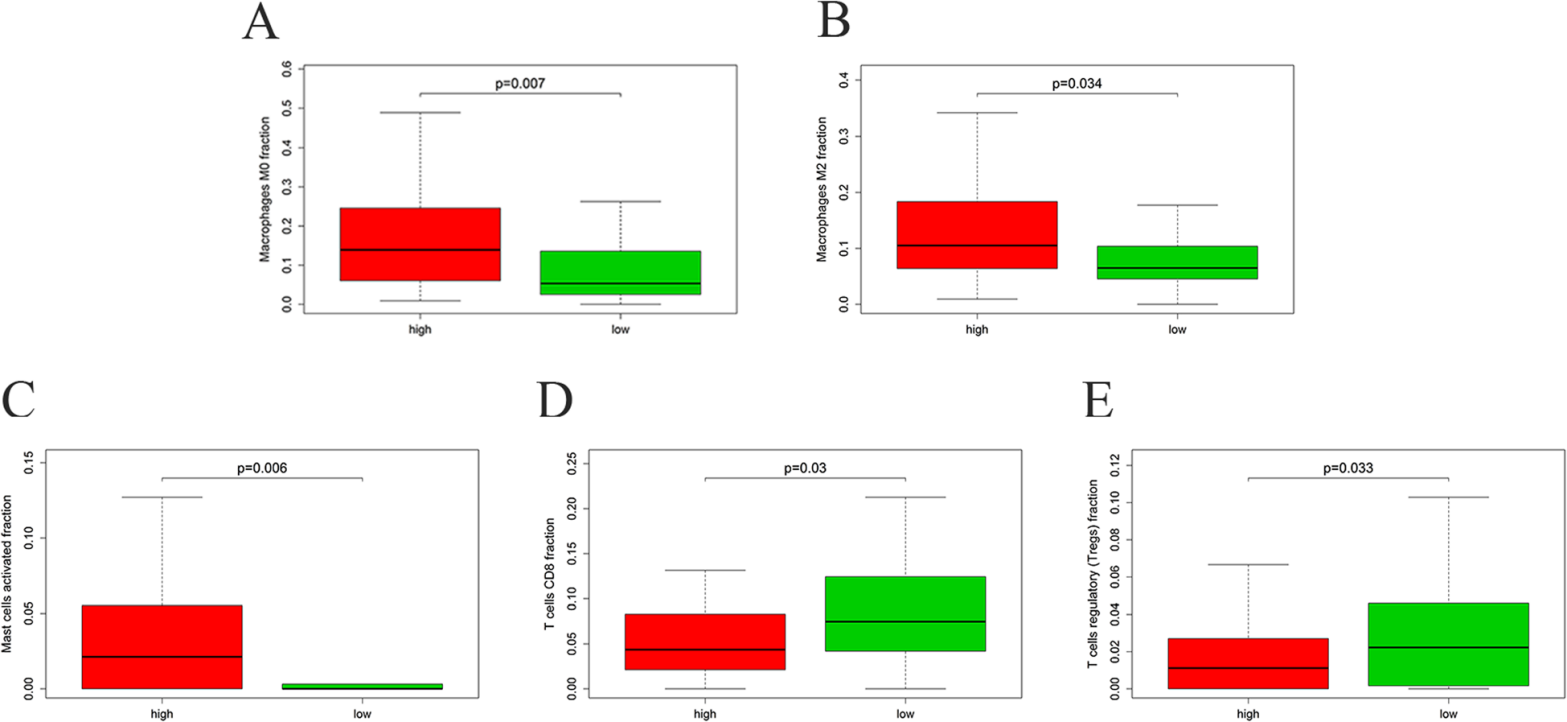
Relationships between the immune-related prognostic index and infiltration abundances of five types of immune cells. **a** Macrophages M0. **b** Macrophages M2. **c** activated mast cells. **d** T cells CD8. **e** T cells regulatory (Tregs); the red parts present high risk group and the green parts represent low risk group

To explore the relationship between the present IRG-based prognostic signature and tumor immune microenvironment, we compared the infiltration of immune cells in different risk groups as defined by the present IRG-based prognostic signature. The results showed that Macrophages M0, Macrophages M2 and activated mast cells were significantly enriched in high-risk group, while CD8 T cells and regulatory T cells (Tregs) were significantly enriched in the low-risk group (Fig. 12). At the same time, there was no significant difference in the enrichment of some other immune subsets between the two groups, such as B cells naive, Eosinophils, Mast cells resting, T cells gamma delta, T cells follicular helper, Plasma cells, NK cells resting, Macrophages M1, B cells memory, Monocytes, T cells CD4 memory resting, Dendritic cells resting, Neutrophils, Dendritic cells activated, T cells CD4 memory activated and NK cells activated (Supplementary Fig. 2).

## Discussion

Esophageal cancer has a large number of new cases every year, and it has historically been regarded as an uncontrollable disease process. The etiology of esophageal cancer may be multifactorial, but part of it is due to the unique manifestation of this cancer [21]. At present, for the treatment of esophageal cancer, attention has shifted to the development of immunotherapy with novel immune biomarkers [22]. Somatic cells acquire malignancy through genetic alterations. Cancer cells usually evade the recognition of the immune system and develop into clinically meaningful masses [23]. Compared with conventional therapies, cancer immunotherapy shows long-lasting response with fewer adverse reactions [24]. This provides a new option for the treatment of EC.

The prognostic model for EC has been continuously updated [25–27]. In this study, we identified 247 up-regulated and 56 down-regulated IRGs in EC and screened out survival-related IRGs. Based on these data, we established a prognostic model that divided EC patients into high-risk and low-risk groups. This model showed a good predictive performance (AUC 0.826). The model was also an independent prognostic indicator by multivariate analysis incorporating other clinical factors. KEGG analysis indicated that the main pathway was enriched in cytokine-cytokine receptor interaction. Many biological processes are regulated by cytokines, including cell growth, differentiation, immunity, inflammation, and metabolism [28]. Tumor progression can be promoted by cytokines that affect the tumor microenvironment and directly act on cancer cells [29]. Moreover, cytokines participate in the immune response of cytotoxic T lymphocytes (CTLs) by modulating the differentiation of Th1 and Th2 cells [30]. Kita Y et al. found that STC2 may be involved in lymph node metastasis, making it a potential prognostic marker for patients with EC [31]. Studies also demonstrated that STC2 may play an important role in ESCC tumorigenesis [32]. Abnormal expression of DKK1, which is regulated by DKK1-CKAP4 pathway, predicts the poor prognosis of esophageal squamous cell carcinoma (ESCC) [33]. These results are consistent with our findings. CacyBP regulates cell proliferation, tumorigenesis, differentiation or gene expression [34]. In colon cancer, CacyBP can promote the growth of cancer cells by enhancing the ubiquitin-mediated degradation of p27kip1 [35]. In addition, studies have confirmed that CacyBP level increased in gastric, nasopharyngeal carcinoma, osteogenic sarcoma and melanoma [36, 37].

In our prognostic model, the IRGs showing prognostic values included HSPA6, S100A12, CACYBP, NOS2, DKK1, OSM, STC2, ANGPTL3 and NR2F2. Among the, HSPA6 may be associated with early recurrence of HCC [38]. In ESCC, S100A12 is downregulated at the protein level [39]. In Barrett’s esophagus and related adenocarcinoma, expression of inducible nitric oxide synthase (NOS-2) is increased, and NOS-2 also plays a role in inflammation and epithelial cell growth [40]. OSM has been identified as an inhibitor of tumor cell growth in a variety of cancers, including melanoma, ovarian cancer, and glioblastoma carcinomas [41–43]. The splice variant of oncostatin M receptor β is overexpressed in human esophageal squamous cell carcinoma [44]. Angiopoietin-like protein 3(ANGPTL3) is indicative of EC prognosis [45]. NR2F2 is involved in the progression of prostate adenocarcinoma [46], and NR2F2 expression is a prognostic factor for breast neoplasms [47]. High expression of NR2F2 in certain gastric and esophageal adenocarcinomas is associated with abnormal expression of cadherin 11, suggesting that the NR2F2-related embryonic pathways in these tumors are reactivated [48]. Proteasome dysregulation is implicated in the development of many types of cancer [49]. The proteasome is involved in cell cycle and transcription, two processes indispensable for cancer development [50]. The spliceosome catalyzes pre-mRNA splicing, a key regulatory step in gene expression [51, 52]. Mutations in genes encoding splice proteins are frequently found in cancer [53]. Small molecule inhibitors that target splice components can be used to create anti-cancer drugs [52]. RNA degradation is a key post-transcriptional regulatory checkpoint to maintain proper functions of organisms. Ribonuclease, a key enzyme responsible for RNA stability, can be used alone for RNA degradation, and can bind to other proteins in the RNA degradation complex [54].

Previous immunotherapies mainly rely on T cells in tumor immune defense [55, 56]. In the present research, the abundance of CD8 T cells and regulatory T cells in the low-risk group increased. T cells are critical in host defense against cancer [57]. The value of CD8 T cells for cancer prognosis has been assessed [58–62]. In addition, CD8 T cells also play a role in the progression of EC [63, 64].

Tregs are divided into two major subpopulations: thymus-derived Tregs (nTregs) and inducible Tregs (iTregs) [65]. Tregs show significant versatility in their inhibitory mechanisms by releasing cytokines to directly inhibit signal transduction of effector T cells [66]. Tregs can also inhibit and kill B cells by inducing programmed cell death [67]. Indeed, Treg infiltration into the tumor has been negatively correlated to OS in a majority of human solid tumors [68, 69]. However, this correlation is highly variable, depending on the tumor type [70]. In cancers that share a common feature of prominent chronic inflammation, such as colon, breast, bladder or head and neck cancers, intra-tumor accumulations of Treg appear to associate with favorable prognosis and improved OS [71–73] .This association has been explained by the capability of Treg to suppress “tumor promoting inflammation” (TPI). Moreover, previous study found that regulatory T cells are positively correlated with locoregional control may be through down-regulation of harmful inflammatory reaction, which could favor tumor progression in head and neck squamous cell carcinoma [71]. So it can be explained that why the abundance of regulatory T cells (Treg) in the low-risk group was higher than in the high-risk group in our finding. In high-risk group, we found that macrophages M0, M2 and activated mast cells were also significantly enriched. Tumor-associated macrophages are the most abundant cancer immune cells. Studies have found that the transcription factor forkhead box protein O1 (FOXO1) can promote the polarization of macrophages M0 to M2 and the recruitment of macrophages M2 in ESCC through transcriptional regulation [74]. Macrophage M2 can be transformed into macrophage M1, and can promote the proliferation, migration and ring-forming ability of lymphatic endothelial cells associated with EC [75]. In addition, macrophage M2 can promote the migration and invasion of ESCC cells, enhance the epithelial-mesenchymal transition process, and promote tumor progression, resulting in poor prognosis for ESCC patients [76]. Tissue kallikrein (TK1), which is highly expressed in activated mast cells, can participate in the formation of mitogenic kinin, which can stimulate the proliferation of tumor cells and enhance metastasis by increasing vascular permeability [77]. All these researches above can support the finding of our study.

It is the first time that a prognostic nomogram is developed with nine immune related genes. This nomogram can be routinely applied and is cost-effective in practice, as it does not need whole-genome sequencing for EC patients. When combined with clinical parameters like TNM stage, the nomogram can show a greater prognostic performance.

Although we constructed a novel nine-gene prognostic signature in esophageal cancer, several limitations of this study should also be acknowledged. Firstly, our prognostic signature was only based on the data from TCGA database, which is not validated in other databases or other centers across different populations. The performance of this prognostic signature might be more reliable if validation is performed with independent external data sets with long-term follow up. Secondly, this study only preliminary proposed a prognostic model and the validity of the five-gene signature model needs to be further verified by clinical trials. Our study was designed on the basis of a retrospective analysis and prospective research should be performed to verify the outcomes. Thirdly, the mechanisms underlying the nine immune-related genes in the prognosis prediction of esophageal cancer needed to be investigated through in vitro and in vivo experiments.

## Conclusions

We identified the IRGs associated with the prognosis of EC and developed an IRGs-based prognostic signature that stratify EC patients into two subgroups with statistically different survival outcomes.

## Supplementary Information

**Additional file 1: Supplementary Fig. 1.** Analysis of the prognosis-related IRGs in box plots by Oncomine. A: HSPA6 mRNA expression (*P*-value:1.10E-10, t-Test:7.750, Fold Change:2.314). B: S100A12 mRNA expression (P-value:0.007, t-Test:2.888, Fold Change:6.630). C: CACYBP mRNA expression (P-value:0.004, t-Test:3.328, Fold Change:1.430). D: NOS2 mRNA expression (P-value:0.010, t-Test:2.485, Fold Change:1.566). E: DKK1 mRNA expression (P-value:0.038, t-Test:1.985, Fold Change:2.128). F: OSM mRNA expression (P-value:3.90E-8, t-Test:5.887, Fold Change: 2.045). G: STC2 mRNA expression (P-value:4.18E-7, t-Test:7.176, Fold Change: 2.293). H: ANGPTL3 mRNA expression (P-value:0.663, t-Test:-0.432, Fold Change:-1.169). I: NR2F2 mRNA expression (P-value:0.974, t-Test:-1.972, Fold Change: -1.318).(1: Barrett’s Esophagus;2: Esophageal Carcinoma; ***P < 0.001; **P < 0.01;*P < 0.05; ns:no significance).

**Additional file 2: Supplementary Fig. 2.** Differences between the immune-related prognostic index and infiltration abundances of other important types of immune cells.

**Additional file 3: Supplementary Table 1.** Characteristics of the dataset.

## Data Availability

The datasets analyzed during the current study are available in the TCGA repository (https://portal.gdc.cancer.gov).

## Abbreviations

EC: Esophageal cancer
IRGs: Immune-related genes
TCGA: The Cancer Genome Atlas
TFs: Transcription factors
Tregs: Regulatory T cells
ESCC: Esophagus Squamous Cell Carcinoma
EA: Esophagus Adenocarcinoma
ImmPort: Immunology Database and Analysis Portal
DEGs: Differentially expressed genes
FDR: False discovery rate
GO: Gene Ontology
KEGG: Kyoto Encyclopedia of Genes and Genomes
ROC: Receiver operating characteristic
AUC: Area under curve
THPA: The Human Protein Atlas
GSEA: Gene Set Enrichment Analysis
BP: Biological Process
CC: Cellular Component
MF: Molecular Function
CTLs: Cytotoxic T lymphocytes
nTregs: Thymus-derived Tregs
iTregs: Inducible Tregs
TPI: Tumor promoting inflammation
FOXO1: Forkhead box protein O1
TK1: Tissue kallikrein
