# Supplementary figures and images for: Development of a prognostic signature for esophageal cancer based on nine immune related genes
Source: BMC Cancer. 2021 Feb 4;21:113. doi: 10.1186/s12885-021-07813-9 (PMC7860013; doi:10.1186/s12885-021-07813-9)

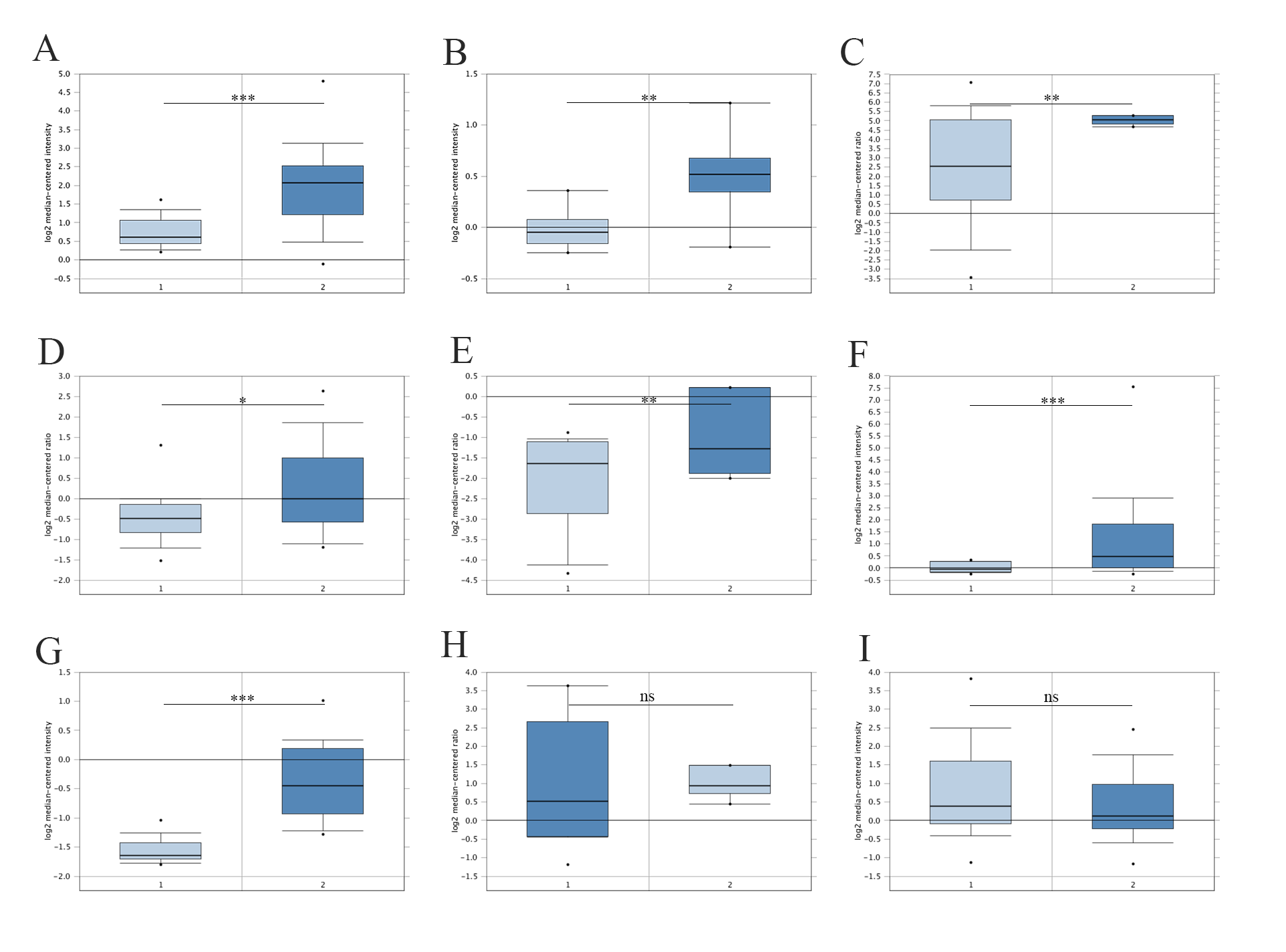

Supplement: Supplementary file 1 — Additional file 1: Supplementary Fig. 1. Analysis of the prognosis-related IRGs in box plots by Oncomine. A: HSPA6 mRNA expression (P-value:1.10E-10, t-Test:7.750, Fold Change:2.314). B: S100A12 mRNA expression (P-value:0.007, t-Test:2.888, Fold Change:6.630). C: CACYBP mRNA expression (P-value:0.004, t-Test:3.328, Fold Change:1.430). D: NOS2 mRNA expression (P-value:0.010, t-Test:2.485, Fold Change:1.566). E: DKK1 mRNA expression (P-value:0.038, t-Test:1.985, Fold Change:2.128). F: OSM mRNA expression (P-value:3.90E-8, t-Test:5.887, Fold Change: 2.045). G: STC2 mRNA expression (P-value:4.18E-7, t-Test:7.176, Fold Change: 2.293). H: ANGPTL3 mRNA expression (P-value:0.663, t-Test:-0.432, Fold Change:-1.169). I: NR2F2 mRNA expression (P-value:0.974, t-Test:-1.972, Fold Change: -1.318).(1: Barrett’s Esophagus;2: Esophageal Carcinoma; ***P < 0.001; **P < 0.01;*P < 0.05; ns:no significance). [file 12885_2021_7813_MOESM1_ESM.tif]

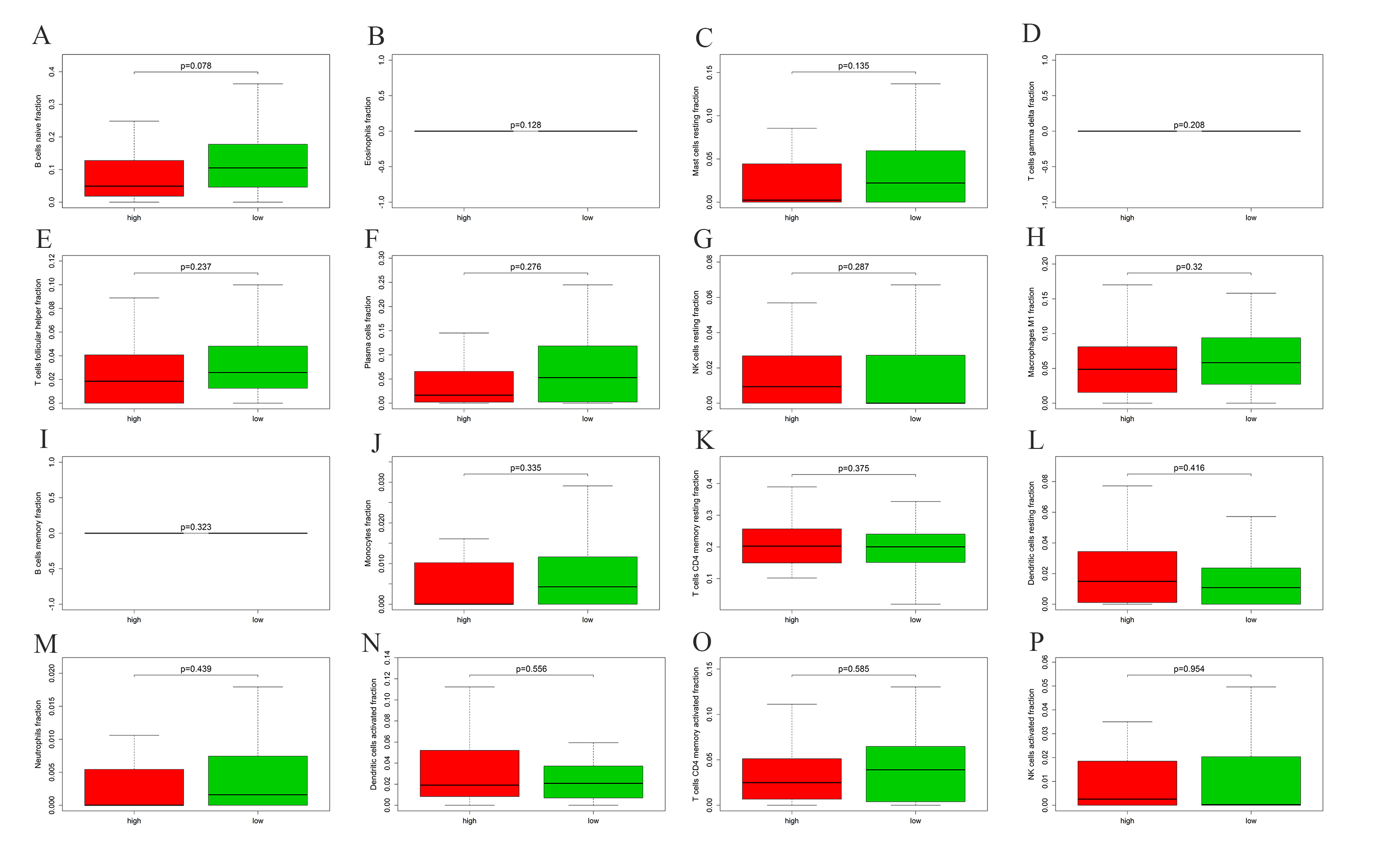

Supplement: Supplementary file 2 — Additional file 2: Supplementary Fig. 2. Differences between the immune-related prognostic index and infiltration abundances of other important types of immune cells. [file 12885_2021_7813_MOESM2_ESM.tif]
